# Exploring contrast-enhanced MRI findings of the clinically non-inflamed symptomatic pediatric wrist

**DOI:** 10.1007/s00247-020-04739-5

**Published:** 2020-07-13

**Authors:** Floris Verkuil, E. Charlotte van Gulik, Charlotte M. Nusman, Anouk M. Barendregt, Amara Nassar-Sheikh Rashid, Dieneke Schonenberg-Meinema, Koert M. Dolman, Mario Maas, Taco W. Kuijpers, J. Merlijn van den Berg, Robert Hemke

**Affiliations:** 1 Pediatric Immunology, Rheumatology and Infectious Diseases, Emma Children’s Hospital, Amsterdam University Medical Centers, Academic Medical Center, University of Amsterdam, Meibergdreef 9, 1105 AZ Amsterdam, The Netherlands; 2 Radiology and Nuclear Medicine, Amsterdam University Medical Centers, Academic Medical Center Amsterdam Movement Sciences, University of Amsterdam, Amsterdam, The Netherlands; 3 Department of Pediatrics, OLVG Oost, Amsterdam, The Netherlands; 4 Department of Pediatrics, OLVG West, Amsterdam, The Netherlands; 5 Pediatric Rheumatology, Reade, Amsterdam, The Netherlands

**Keywords:** Adolescents, Children, Contrast enhancement, Juvenile idiopathic arthritis, Magnetic resonance imaging, Synovial membrane, Wrist joint

## Abstract

**Background:**

Knowledge of the synovial and tenosynovial appearance of the clinically non-arthritic symptomatic juvenile wrist using contrast-enhanced magnetic resonance imaging (MRI) is sparse.

**Objectives:**

To analyze contrast-enhanced MRI findings of the clinically non-inflamed symptomatic pediatric wrist, focusing on the enhancing synovial and tenosynovial membrane. To evaluate the coexistent presence of (teno)synovial enhancement, joint fluid, bony depressions and medullary changes suggestive of bone marrow edema.

**Materials and methods:**

We included 20 children (15 girls; age range: 7.5–17.6 years) who underwent contrast-enhanced MRI of the wrist, based on initial clinical indication, and eventually turned out to be unaffected by arthritic or orthopedic disorders. Various imaging characteristics of the synovium, tenosynovium, joint fluid, bone tissue and bone marrow were evaluated using existing MRI scoring systems.

**Results:**

In 3/20 (15%) children, mild or moderate-severe synovial enhancement was observed and 2/20 (10%) children showed mild tenosynovial enhancement/thickening. Joint fluid (11/20 children; 55%), bony depressions (20/20 children; 100%) and medullary changes suggestive of bone marrow edema (6/20; 30%) were found in a substantial percentage of children. The most frequently observed combination of coexisting imaging characteristics was bony depressions with ≥2 mm joint fluid, which was found in 7/20 (35%) children. Simultaneous presence of synovial and tenosynovial enhancement/thickening, bony depressions and medullary changes suggestive of bone marrow edema was observed in one child.

**Conclusion:**

Several juvenile idiopathic arthritis-relevant MRI characteristics can be observed in the clinically non-inflamed symptomatic pediatric wrist.

**Electronic supplementary material:**

The online version of this article (10.1007/s00247-020-04739-5) contains supplementary material, which is available to authorized users.

## Introduction

Important steps contributing to a magnetic resonance imaging (MRI) atlas of the healthy pediatric wrist have been made [1–4]. For example, it is shown that bony depressions and joint fluid can be found in the wrist joint in a substantial percentage of healthy children [1, 2]. In children with juvenile idiopathic arthritis, contrast-enhanced MRI is the preferred imaging technique to evaluate synovial inflammation [5–7], which is the hallmark of the disease’s activity [8]. Despite the increasing knowledge about imaging features of the healthy juvenile wrist [1–4], reference standards for the non-inflamed appearance of the enhancing synovium are sparse, which hampers the use of contrast-enhanced MRI in the diagnostic process of juvenile idiopathic arthritis. The contrast-enhanced synovial membrane in children clinically unaffected by inflammatory pathology has only been assessed in the knee joint [9]. As summarized by Ejbjerg et al. [10], several studies evaluated the enhancing synovial membrane of the wrist joint in healthy adults. However, normal values as used in the adult population are not automatically applicable to children, since similar MRI findings in both groups might represent different processes. For example, signal changes suggestive of bone marrow edema, which is associated with rheumatoid arthritis activity in adults [11, 12], have been detected in the wrist and knee joint of healthy children and might be related to the physiological process of maturation [1, 2, 9].

To contribute to the process of establishing reference standards to evaluate the wrist joint in children with suspected juvenile idiopathic arthritis, the aim of this study is to assess the enhancing synovial and tenosynovial membrane of the clinically non-inflamed symptomatic pediatric wrist using contrast-enhanced MRI. Secondly, we evaluate the coexistent presence of (teno)synovial enhancement/thickening, joint fluid, bony depressions and medullary changes suggestive of bone marrow edema.

## Materials and methods

### Patients

From December 2008 until February 2018, all children with clinically active disease (suspected juvenile idiopathic arthritis, new-onset juvenile idiopathic arthritis or remitting/relapsing juvenile idiopathic arthritis with wrist involvement) or clinically inactive disease (medical history of juvenile idiopathic arthritis with wrist involvement) who underwent contrast-enhanced MRI examination of the wrist joint were prospectively included. At the time of presentation, all included patients subsequently underwent clinical and laboratory assessment, followed by contrast-enhanced MRI of the wrist based on clinical indication. This multicenter registry was conducted in three pediatric rheumatology centers: Amsterdam University Medical Centers (Academic Medical Centre location), Reade and Onze Lieve Vrouwe Gasthuis, all in Amsterdam, the Netherlands.

**Fig. 1:**
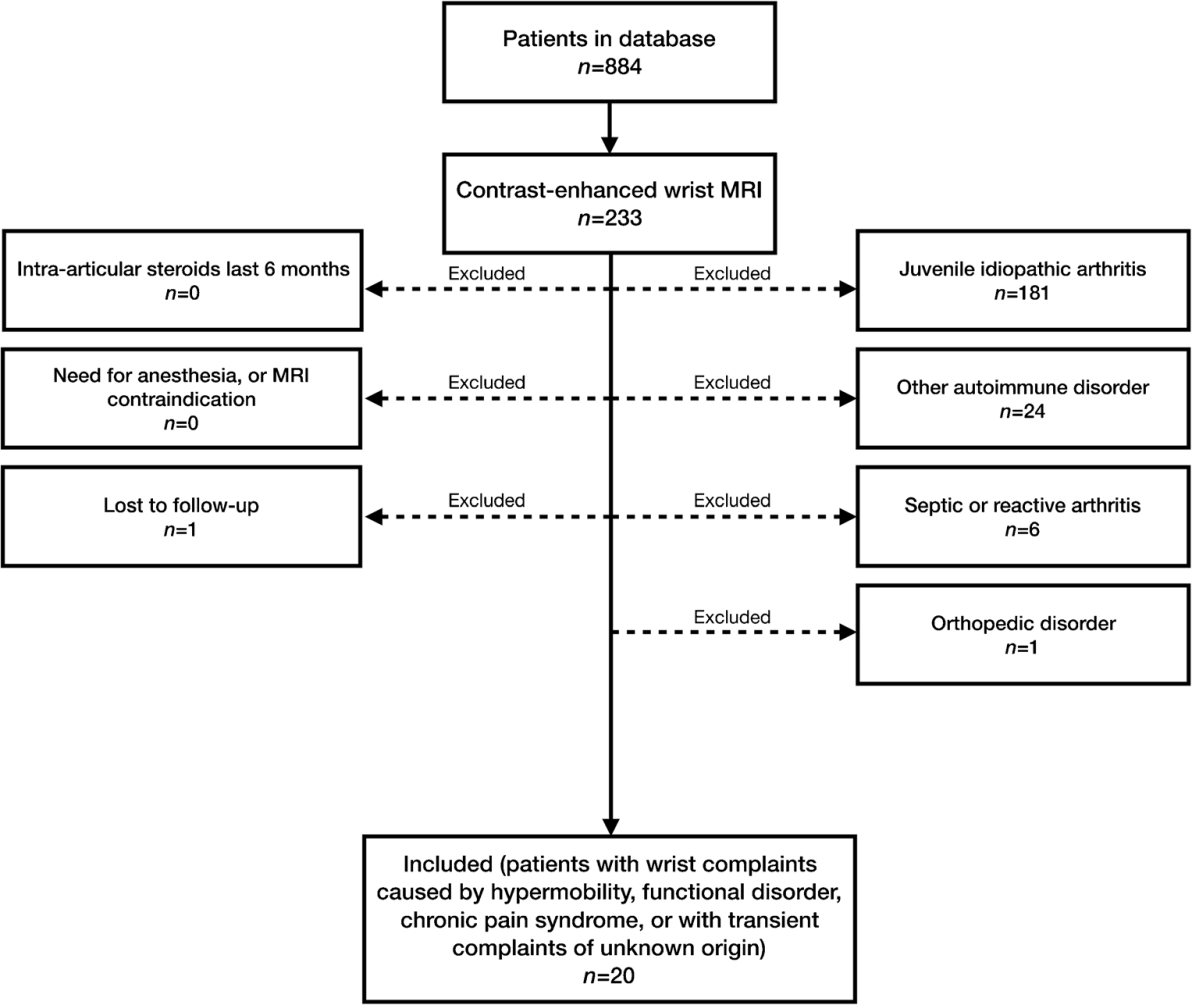
Flowchart: patient inclusion

For the purpose of this study, we included children who turned out to be clinically unaffected by arthritis-related diseases (i.e. any disorder potentially resulting in [accompanying] arthritis) or orthopedic disorders after follow-up. All consecutive children were included in whom the final diagnosis was: (1) joint hypermobility, (2) a functional disorder/chronic pain syndrome or (3) transient wrist complaints of unknown origin. The exclusion criteria were: (1) diagnosis of juvenile idiopathic arthritis according to the International League of Associations for Rheumatology criteria [13], (2) diagnosis of an autoimmune disorder (other than juvenile idiopathic arthritis) that may possibly result in arthritis, (3) arthritis secondary to an infection (i.e. septic arthritis or reactive arthritis), (4) wrist complaints caused by an orthopedic disorder, (5) an intra-articular injection of corticosteroids within the last 6 months, (6) the need for anesthesia-assisted MRI examination and (7) the principal contraindications for MRI. The study flowchart is depicted in Fig. 1. The local ethics committee waived informed consent for this study.

### Clinical and laboratory assessment

According to the International League of Associations for Rheumatology criteria [13], the eventual decision to confirm or reject the diagnosis of juvenile idiopathic arthritis was made after a follow-up period of at least 6 months by 1 of the 4 experienced pediatric rheumatologists (A.N.-S.R., with 3 years of experience, D.S.-M., with 7 years of experience, J.M.v.d.B., with 13 years of experience, and K.M.D., with 16 years of experience). Clinical evaluation consisted of a joint-specific assessment of swelling, warmth, motion discomfort and range of motion for 71 joints. Laboratory tests included the erythrocyte sedimentation rate and level of C-reactive protein. Additional measurements of disease activity consisted of the Dutch version of the Childhood Health Assessment Questionnaire, the physician’s global assessment of overall disease activity, the patient’s global assessment of general health and the patient’s global assessment of pain; all (except for the Childhood Health Assessment Questionnaire) were scored on a 100-mm visual analogue scale.

### Magnetic resonance imaging protocol

MRI examinations were performed with an open bore 1.0-tesla (T) MR scanner (Panorama HFO; Philips Medical Systems, Best, the Netherlands) from December 2008 to January 2015 and a 3.0-T MR scanner (Ingenia; Omega, Philips Medical Systems) from February 2015 until February 2018. Both scanners were equipped with a dedicated wrist coil. All patients were placed in a feet-first supine position with the arm in neutral position and the wrist joint placed in the middle of the wrist-coil. The wrist most clinically involved was considered to be the target joint for MRI. In case there were no differences in clinical disease activity between both wrists, the right side was selected as the target joint. The following sequences were acquired for the 1.0-T MR scanner: coronal and transversal T1-weighted images, coronal T1-weighted fat-saturated images, transversal T2-weighted fat-saturated images, coronal T1-weighted post-contrast images and transversal T1-weighted fat-saturated post-contrast images. For the 3.0-T MR scanner, sequences included: transversal T1-weighted turbo spin echo images, coronal and transversal proton density weighted images, coronal T1-weighted Dixon pre- and post-contrast images and transversal T1-weighted fat-saturated post-contrast images. To standardize the timing of post-contrast image acquisition, and hereby diminish the influence of time-dependent variability in the degree of (teno)synovial enhancement [14], and to improve distinction between synovial enhancement and joint fluid [15], all post-contrast images were obtained <5 min after intravenous gadolinium (0.1 mg/kg of body weight; gadobutrol, Schering AG, Berlin, Germany) was administered.

### Image analysis

To avoid any bias, MR images of the included children were randomly added to a larger dataset of MR images of patients diagnosed with juvenile idiopathic arthritis and all scored in consensus by 2 experienced readers (R.H. and M.M., with 9 and 21 years of experience in musculoskeletal MRI, respectively) blinded for patient characteristics and clinical data.

All imaging features of interest were evaluated in accordance with existing scoring systems [1, 16–19]. The level of synovial enhancement (0=normal, 1=mild, 2=moderate-severe) was assessed in 5 predefined anatomical locations and graded according to the scoring system designed by Damasio et al. [16]. For the assessment of the level of tenosynovial enhancement and thickening (0=normal, 1=mild, 2=moderate), evaluated in 4 different tendon compartments of the wrist, we used the tenosynovitis MRI scoring system developed by Lambot et al. [17]. The volume of joint fluid (0=not visible, 1=>0 mm to <2 mm, 2=≥2 mm) was scored in 12 predefined joints according to the scoring system as used in the study by Ording Müller et al. [1]. The number of bony depressions was scored in 12 predefined locations according to the template presented by Avenarius et al. [18]. Medullary changes suggestive of bone marrow edema were evaluated in 14 areas and scored as the estimated percentage of bone that is edematous (0=no bone marrow edema, 1=1–33%, 2=34–66%, 3=67–100%), according to the MRI scoring system developed by Tanturri de Horatio et al. [19]. For an overview of the used scoring systems, see Table 1.

**Table 1:** Overview of the scoring systems (including predefined locations and grading methods)

MRI features	Locations^a^	Grading	Total score^b^
Contrast-enhanced synovial membrane [16]	1. Distal radioulnar 2. Radiocarpal 3. Midcarpal 4. 1^st^ CMC 5. 2^nd^–5^th^ CMC	*Synovial enhancement (grading scale: 0–2)* 0=normal 1=mild enhancement 2=moderate-severe enhancement	Range: 0–10
Contrast-enhanced tenosynovial membrane [17]	1. 2^nd^ dorsal extensor tendon compartment^c^ 2. 4^th^ dorsal extensor tendon compartment^d^ 3. 6^th^ dorsal extensor tendon compartment^e^ 4. Carpal tunnel^f^	*Tenosynovial enhancement/thickening (grading scale: 0–2)* 0=normal tendon sheath enhancement/no thickening 1=tendon sheath enhancement/mild thickening 2=tendon sheath enhancement/moderate thickening	Range: 0–8
Joint fluid [1]	1. Radius/scaphoid 2. Radius/lunate 3. Radioulnar 4. Trapezium/scaphoid 5. Capitate/hamate 6. Hamate/triquetrum 7. Hamate, laterally 8. Pisotriquetral	*Joint fluid (grading scale: 0–2)* 0=not visible 1= >0 mm to <2 mm 2= ≥2 mm	NA

*CMG* carpometacarpal, *NA* not applicable

^a^In accordance with the scoring system developed by Tanturri de Horatio et al. [19], we evaluated the radius, ulna and metacarpal bases (i.e. from the articular surface to a depth of 1 cm)

^b^Total scores were obtained by summing the scores for the assessed anatomical locations

^c^Extensor carpi radialis brevis and extensor carpi radialis longus

^d^Extensor digitorum communis and extensor indicis proprius

^e^Extensor carpi ulnaris

^f^Flexor digitorum superficialis and the flexor digitorum profundus tendons

### Statistics

Descriptive statistics were reported in terms of absolute numbers, percentages, medians and interquartile ranges. In conformity with Ording Müller et al. [1], the number of bony depressions was subgrouped based on total scores (i.e. 0=<5 depressions, 1=5–10 depressions and 2=>10 depressions). To compare numerical and categorical endpoints between the three included subgroups, we used the Kruskal-Wallis test and the chi-square test, respectively. Statistical significance was defined as a two-tailed *P*-value <0.05.

## Results

### Patients

We included and analyzed data of 20 children (15/20 [75%] girls) with a median age of 14.7 years (age range: 7.5–17.6 years). An overview of clinical characteristics is shown in Table 2. Of the included children, 13/20 (65%) had transient wrist complaints of unknown origin, 5/20 (25%) had joint complaints based on a functional disorder/chronic pain syndrome and 2/20 (10%) had wrist complaints due to joint hypermobility.

**Table 2:** Baseline characteristics of the 20 patients

Female, *n* (%)	15 (75.0)
Age at study visit (years), median (IQR)	14.7 (12.6–16.8)
Physician’s global assessment of overall disease activity (VAS), median (IQR)	14.0 (10.0–19.5)
Patient’s global assessment of overall well-being (VAS) (*n*=14), median (IQR)	50.0 (43.5–60.0)
Patient’s pain assessment (VAS); (*n*=13), median (IQR)	73.0 (28.0–80.0)
Childhood Health Assessment Questionnaire^a^ (*n*=17), median (IQR)	1.3 (0.8–1.8)
C-reactive protein (mg/L), median (IQR)	0.4 (0.0–1.2)
Erythrocyte sedimentation rate (mm/h), median (IQR)	5.0 (2.0–7.5)

*IQR* interquartile range, *VAS* visual analogue score (0=best, 100=worst)

^a^Units: 0=best, 3=worst

### Imaging

Patient characteristics and MRI results did not differ significantly between the three included subgroups.

**Fig. 2:**
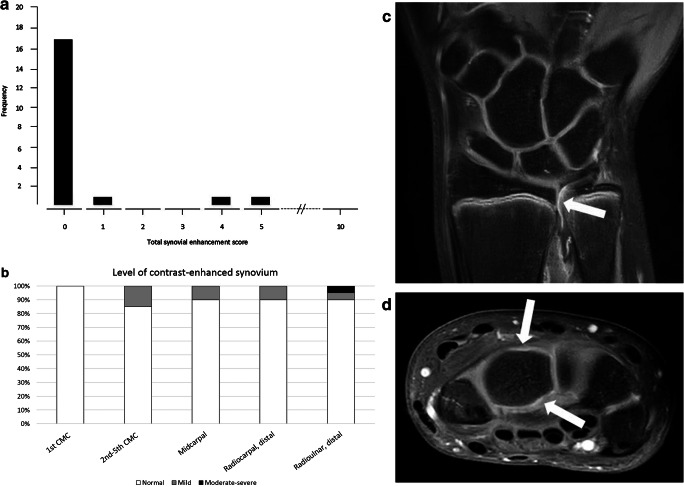
The contrast-enhanced synovial membrane. **a** Overview of the frequency of total synovial enhancement scores. **b** Overview of the percentage of children in whom that particular anatomical location was affected as part of the sum of all included children. **c** Coronal T1-weighted fat-saturated post-contrast image obtained in a 13-year-old girl gives an example of normal synovial enhancement (*arrow*) in the distal radioulnar joint. **d** Axial T1-weighted fat-saturated post-contrast image obtained in a 14-year-old girl gives an example of normal synovial enhancement (*arrows*) in the midcarpal joint. *CMC* carpometacarpal

The level of synovial enhancement was scored as being normal (i.e. grade= 0) in all the assessed anatomical locations in 17/20 (85%) children. The median of the total synovial enhancement score was 0.0 (interquartile range [IQR]: 0.0–0.0), ranging up to 5 as maximum score (*n*=1; a 12-year-old girl with transient wrist complaints of unknown origin) (Fig. 2). A synovial enhancement score of ≥1 was seen in 3/20 (15%) children in one or more anatomical locations. The 2nd-5th carpometacarpal joint was affected in all three children. Only one of the three children (same individual as described above) showed moderate-severe synovial enhancement (i.e. grade=2), located in the distal radioulnar joint (Fig. 2). The 1st carpometacarpal joint was scored as being normal in all the included children. Examples of the normal enhancing synovial membrane are shown in Fig. 2.

**Fig. 3:**
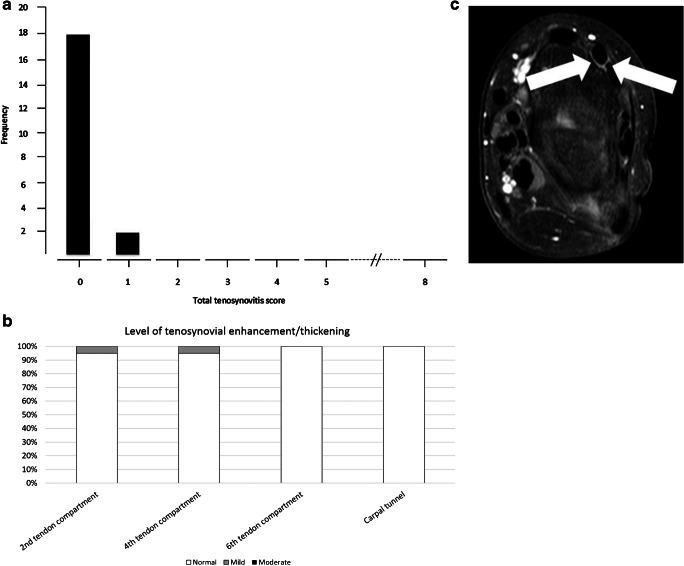
The contrast-enhanced tenosynovial membrane. **a** Overview of the frequency of total tenosynovial enhancement/thickening scores. **b** Overview of the percentage of children in whom that particular anatomical location was affected as part of the sum of all included children. **c** Axial T1-weighted fat-saturated post-contrast image in a 15-year-old boy gives an example of tenosynovial enhancement and mild thickening (*arrows*) in second wrist compartment

In 18/20 (90%) children, the tenosynovium was scored as being normal (i.e. grade= 0). The median of the total score was 0.0 (IQR: 0.0–0.0). The maximum total score was 1 (*n*=2) (Fig. 3). Enhancement and mild thickening of the tendon sheath (i.e. grade 1) was found in 2/20 (10%) children, located in the second (*n*=1) and fourth (*n*=1) dorsal extensor tendon compartment (Fig. 3). For an example of tenosynovial enhancement and thickening, see Fig. 3.

**Fig. 4:**
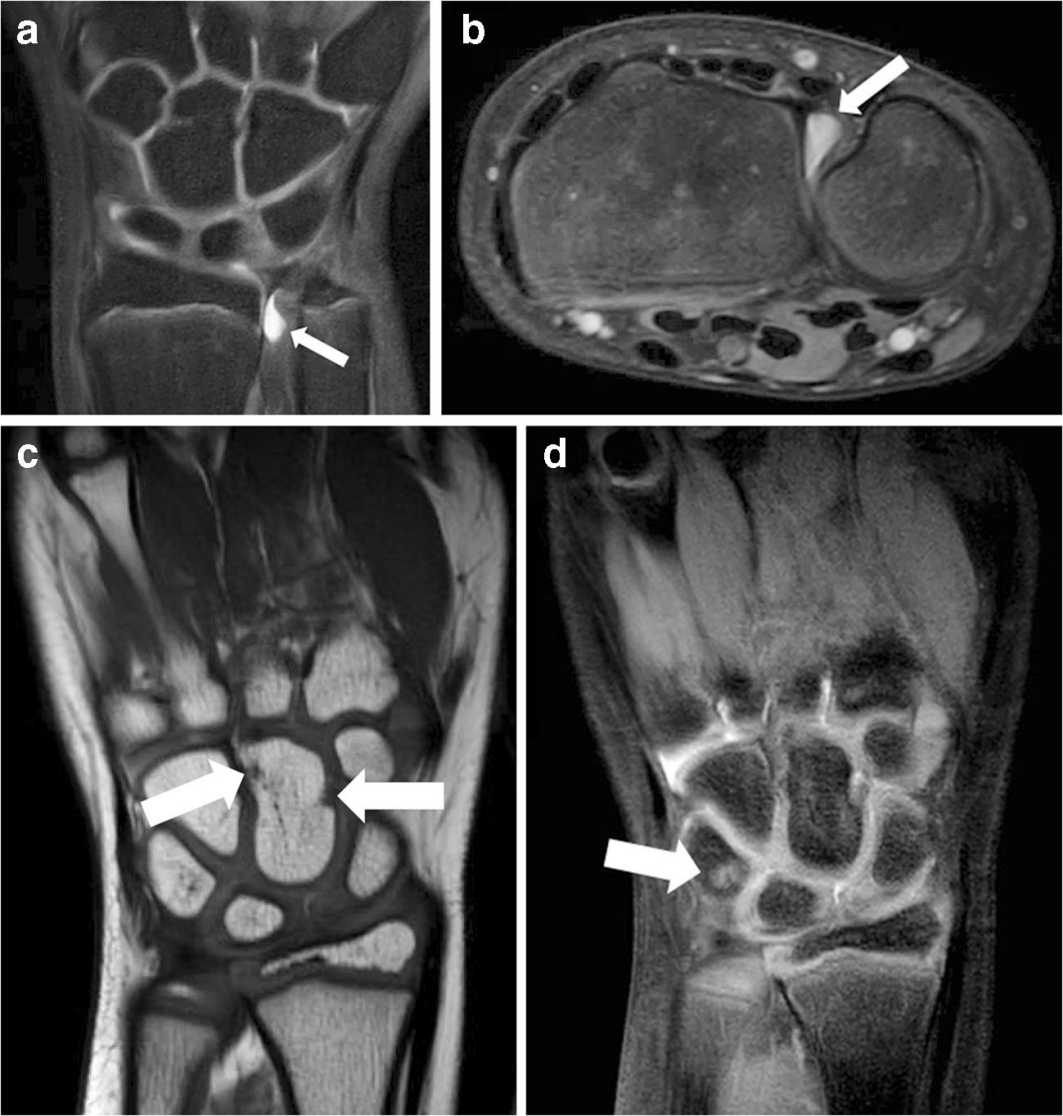
Examples of joint fluid, bony depressions and medullary changes suggestive of bone marrow edema. **a**, **b** Coronal (**a**) and axial (**b**) T2-weighted fat-saturated images in a 13-year-old girl show joint fluid (*arrows*) in the distal radioulnar joint. **c** Coronal T1-weighted mDixon in-phase image in a 7-year-old girl shows bony depressions (*arrows*) in the capitate. **d** Coronal single-point Dixon (sense: water) image in a 7-year-old girl shows signal changes suggestive

All children showed some joint fluid in at least one of the assessed anatomical locations. Moderate volume of joint fluid (i.e. ≥2 mm) was observed in 11/20 (55%) children. Moderate joint fluid was found in seven different locations and most frequently present in the radioulnar, radioscaphoid, pisotriquetral and 1st carpometacarpal joint (Online Supplementary Material 1). Examples of moderate joint fluid are depicted in Fig. 4.

Bony depressions were observed in all 20 included children. The median total number of bony depressions was 9.0 ([IQR: 6.3–9.0). All children had visible bony depressions in the capitate (median number of bony depressions: 3.0 [IQR: 2.0–3.0]) (Online Supplementary Material 2). None of the children showed bony depressions in the 1st metacarpal. None of the children showed bony depressions situated at the metacarpal surface of the carpometacarpal joints. An example of bony depressions is shown in Fig. 4.

Signal changes suggestive of bone marrow edema were present in 6/20 (30%) children. The median of the total score was 0.0 (IQR: 0.0–0.8), ranging up to 2 as maximum total score (*n*=1) (Online Supplementary Material 3). Medullary changes suggestive of bone marrow edema were found in the lunate, triquetrum, trapezium and most frequently in the capitate (Online Supplementary Material 4). For an example of medullary changes suggestive of bone marrow edema, see Fig. 4.

**Fig. 5:**
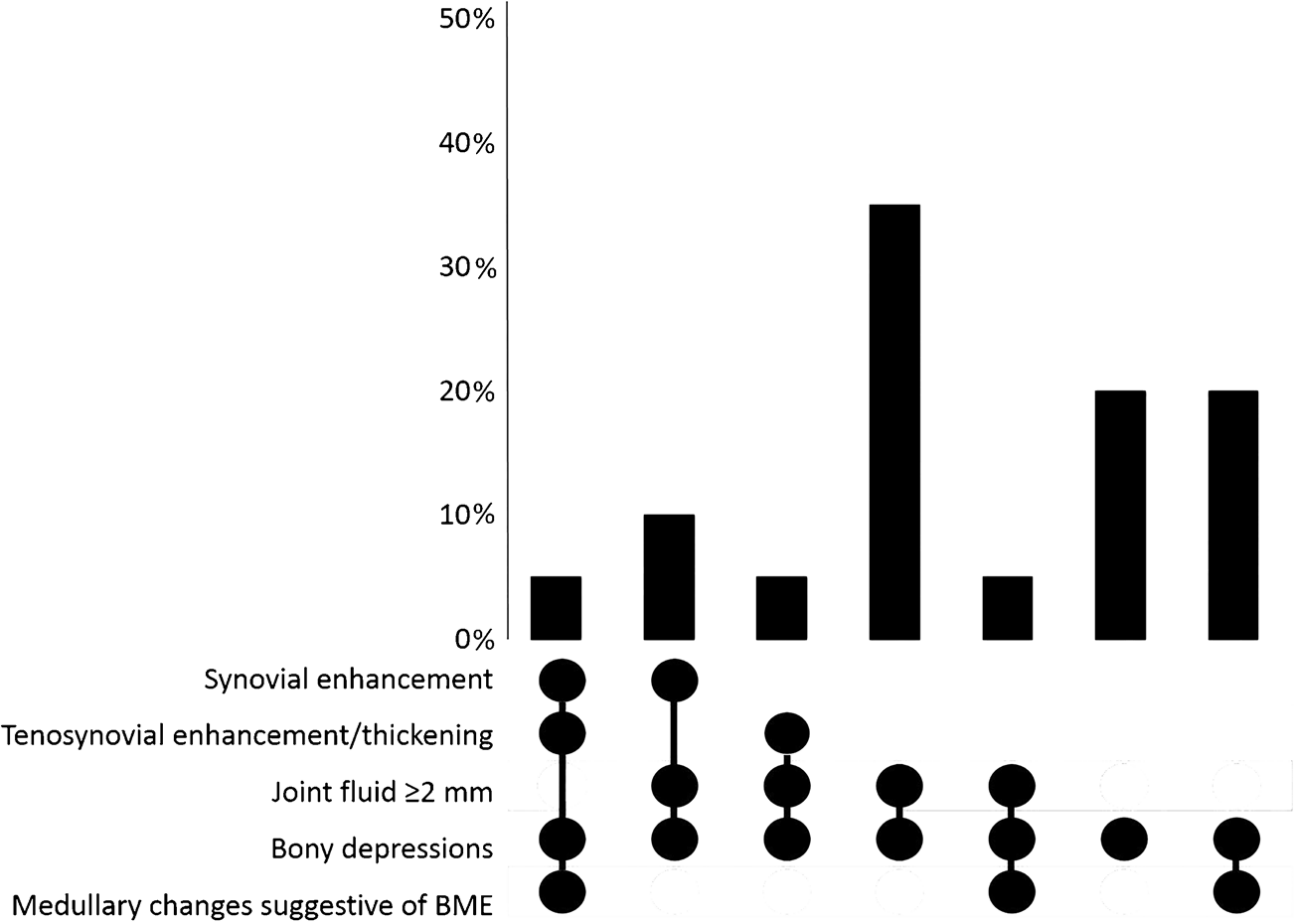
Overview of the combinations of MRI features in the clinically non-inflamed symptomatic wrist joints. This figure shows the percentage of children in whom a given combination of MRI characteristics was present. Only observed combinations are displayed. *BME* bone marrow edema

The context of the incidence per feature is displayed in Fig. 5, which shows the different combinations of coexistence of the assessed MRI characteristics. The simultaneous presence of some synovial enhancement and mild tenosynovial enhancement/thickening bony depressions and medullary changes suggestive of bone marrow edema was observed in one child (the same individual as described above). The coexistent presence of bony depressions and joint fluid ≥2 mm without other features was the most frequently found combination, namely in 7/20 (35%) children.

## Discussion

This study provides new insights in the appearance of the enhancing (teno)synovial membrane of the clinically non-inflamed symptomatic pediatric wrist. We showed that the (teno)synovium was scored as normal in the majority of included children, but mild to moderate-severe synovial enhancement and mild tenosynovial enhancement/thickening can be found in the growing wrists of children with joint complaints that do not originate from inflammatory or orthopedic disease. Additionally, we confirmed that joint fluid, bony depressions and medullary changes suggestive of bone marrow edema are present in a substantial percentage of children unaffected by arthritis-related disorders. The current study further expands the knowledge of the clinically non-inflamed symptomatic juvenile wrist by giving an overview of the different combinations of coexistence of the relevant MRI features.

To evaluate the enhancing synovial membrane, we used the pediatric MRI-based synovitis scoring system as presented by Damasio et al. [16]. In the study by Damasio et al. [16], children diagnosed with juvenile idiopathic arthritis showed a mean total synovial enhancement score of 3 and 4 out of 10 (for readers 1 and 2, respectively). In our study, the level of synovial enhancement was scored as being normal (i.e. grade=0) in all the assessed anatomical locations in the majority (85%) of included children unaffected by inflammatory disease. The median of the total synovial enhancement score was 0. The maximum total score observed was 5, which was found in only one child. Since no total scores above 5 were measured in the remaining children, this could be considered as a potential cutoff value in the diagnostic process of juvenile idiopathic arthritis. However, future studies with large cohorts of children unaffected by arthritic disorders are necessary to corroborate or reject this potential cutoff value. Additionally, our results might indicate that the pediatric MRI synovitis scores of children with juvenile idiopathic arthritis and children with wrist complaints without inflammatory origin could overlap to some extent. For this reason, one should be aware of the possible existence of this grey area while interpreting the level of synovial enhancement in children with suspected juvenile idiopathic arthritis.

We found that 2/20 (10%) children unaffected by arthritic disorders showed some enhancement and mild thickening of the tendon sheath located in the second (*n*=1) and fourth (*n*=1) dorsal extensor tendon compartment. We hypothesize that the tenosynovial enhancement and thickening in the included children could be explained by cumulative microtrauma secondary to local mechanical stress factors (e.g., repetitive movements, muscle overuse, etc.), a cause associated with tenosynovitis [20, 21]. It is difficult to exclude local mechanical stress influencing outcome measurements, since repetitive-motion movements of the hand/wrist tendons and muscles (e.g., text messaging, pressing keys/buttons on a computer keyboard/controller, etc.) are fully integrated in daily activities of the youth [22, 23]. Other explanations for our results could be that tenosynovial enhancement/thickening originate from the noninflammatory wrist complaints or that it is a physiological appearance in the maturation process of the juvenile wrist. Nevertheless, future studies are necessary to confirm the true origin of tenosynovial enhancement/thickening in the clinically non-inflamed symptomatic pediatric wrist.

Damasio et al. [16] showed that joint fluid ≥2 mm, assessed in 6 locations, varied from approximately 12% to 38% in the wrist of children affected by juvenile idiopathic arthritis. We found that all included children showed some joint fluid in at least one of the joints analyzed. Moreover, we found a moderate volume of joint fluid (i.e. ≥2 mm) in 55% of the children. These results coincide with the findings of the study by Ording Müller et al. [1] on a large cohort of 84 healthy Norwegian children who underwent non-contrast enhanced MR imaging of the wrist. Our findings support the theory that joint effusion ≥2 mm, an amount suggested as pathological in the adults [24], might be considered a physiological appearance associated with normal maturation of the healthy juvenile wrist.

In accordance with a study by Ording Müller et al. [1], we evaluated all bony indentations other than the normal vascular channels on T1-weighted images. In our cohort, the total number of bony depressions ranged from 4 to 12 per child (median: 9). These findings coincide with the results of Avenarius et al. [18], who showed a mean total number of bony depressions that varied from 11 in a group ages 10–12 years to 15 in a group ages 16–19 years in a follow-up study of a large cohort of healthy Norwegian children. The authors also demonstrated that most bony depressions were observed in the capitate, followed by the metacarpals and lunate, which conforms with our study. In accordance with the study by Ording Müller et al. [3], in which the authors compared the location of bony depressions between juvenile idiopathic arthritis patients and healthy children, none of the included children in our cohort showed bony depressions situated at the metacarpal surface of the carpometacarpal joints. Therefore, bony indentations located at this specific area might be exclusively seen in children diagnosed with arthritic disease [3]. Nevertheless, as there is still no validated guideline to distinguish a benign bony depression from a pathological bone erosion and the location of bony indentations in juvenile idiopathic arthritis patients, except for the metacarpal surface of the carpometacarpal joints, which are almost similar to those in the healthy pediatric population [3], all bony indentations found in the juvenile wrist should be interpreted with care.

We showed that 30% of our cohort had signs suggestive of bone marrow edema in at least one of the assessed anatomical areas. This high prevalence was not unexpected as our results generally conform to the findings of the previously mentioned study by Ording Müller et al. [1]. That study showed that 53.6% of the included healthy children had MRI signal changes suggestive of bone marrow edema [1]. Altogether, it is unlikely that signal changes suggestive of bone marrow edema without local coexistence of juvenile idiopathic arthritis-relevant MRI features are always related to inflammatory pathology and might also be observed as part of normal maturation.

Nusman et al. [25]. showed that synovial hypertrophy and bone marrow edema were observed simultaneously in the wrist joint of 27.9% of the included children diagnosed with juvenile idiopathic arthritis. In this study, we showed that coexistent synovial and tenosynovial enhancement and medullary changes suggestive of bone marrow edema could also be found in the clinically non-arthritic symptomatic juvenile wrist. Accordingly, these findings, although observed in only one patient, might indicate that the clinically non-inflamed symptomatic pediatric wrist could mimic the contrast-enhanced MRI signature of the arthritic juvenile wrist to some extent. Therefore, the coexistence of (teno)synovial enhancement and medullary changes suggestive of bone marrow edema, without the local presence of other relevant features, might not be sufficient to distinguish inflammatory causes from non-arthritic wrist complaints. However, the simultaneous presence of (teno)synovial enhancement and medullary changes suggestive of bone marrow edema as observed in our study were spread out over the different assessed anatomical areas in the wrist joint and were not locally connected to each other. For this reason, local anatomical connections between simultaneously present relevant MRI features could hypothetically be more valuable to differentiate between arthritic and noninflammatory juvenile wrist pathology. Nevertheless, future studies are necessary to confirm or reject this hypothesis.

Our study has several limitations. The restricted number of children in this study may limit the preciseness of the defined values and may restrict adequate subgroup analyses. Furthermore, the study cohort comprised 20 children between ages 7.5 and 17.6 years; hence, the findings of this study are not representative of the youngest category of children. However, owing to the invasiveness of contrast administration, the use of contrast-enhanced MR imaging in children without a strict clinical indication and in very young children is restricted. For this reason, the inclusion of large cohorts of children at all age stages is difficult. Thirdly, the start of post-contrast data acquisition was not completely standardized, as the images were obtained <5 min after contrast administration. Therefore, there might be some small time-dependent enhancement variability, which could affect the preciseness of the results [14, 15]. Fourthly, X-ray examination of the hand/wrist at the moment of contrast-enhanced MRI was not included in the study protocol. Therefore, we were not able to assess the extent of skeletal maturation adequately, and subsequently were restricted to integrate skeletal maturity in data analyses. Lastly, due to the absence of one validated juvenile arthritis MRI scoring system for the wrist joint, we were obligated to use different scoring systems [1, 16–19] to assess all key imaging characteristics involved in juvenile idiopathic arthritis. To the best of our knowledge, we selected the most validated and suitable scoring systems available in current literature.

## Conclusion

In the majority of included children with clinically non-inflamed symptomatic wrists, the enhancing synovial and tenosynovial membrane was scored as normal, but mild and moderate-severe synovial enahancement, and mild tenosynovial enhancement/thickening could be observed. Given the possibility of the simultaneous presence of synovial enhancement, tenosynovial enhancement/thickening and medullary changes suggestive of bone marrow edema, caution is required when interpreting CE-MRI of the wrist joint of children with suspected juvenile idiopathic arthritis.

## Electronic supplementary material

ESM 1 (JPG 965 kb)

ESM 2 (JPG 74 kb)

ESM 3 (JPG 79 kb)

ESM 4 (JPG 85 kb)
