# Supplementary figures and images for: Exploring contrast-enhanced MRI findings of the clinically non-inflamed symptomatic pediatric wrist
Source: Pediatr Radiol. 2020 Jul 13;50(10):1387–96. doi: 10.1007/s00247-020-04739-5 (PMC7445206; doi:10.1007/s00247-020-04739-5)

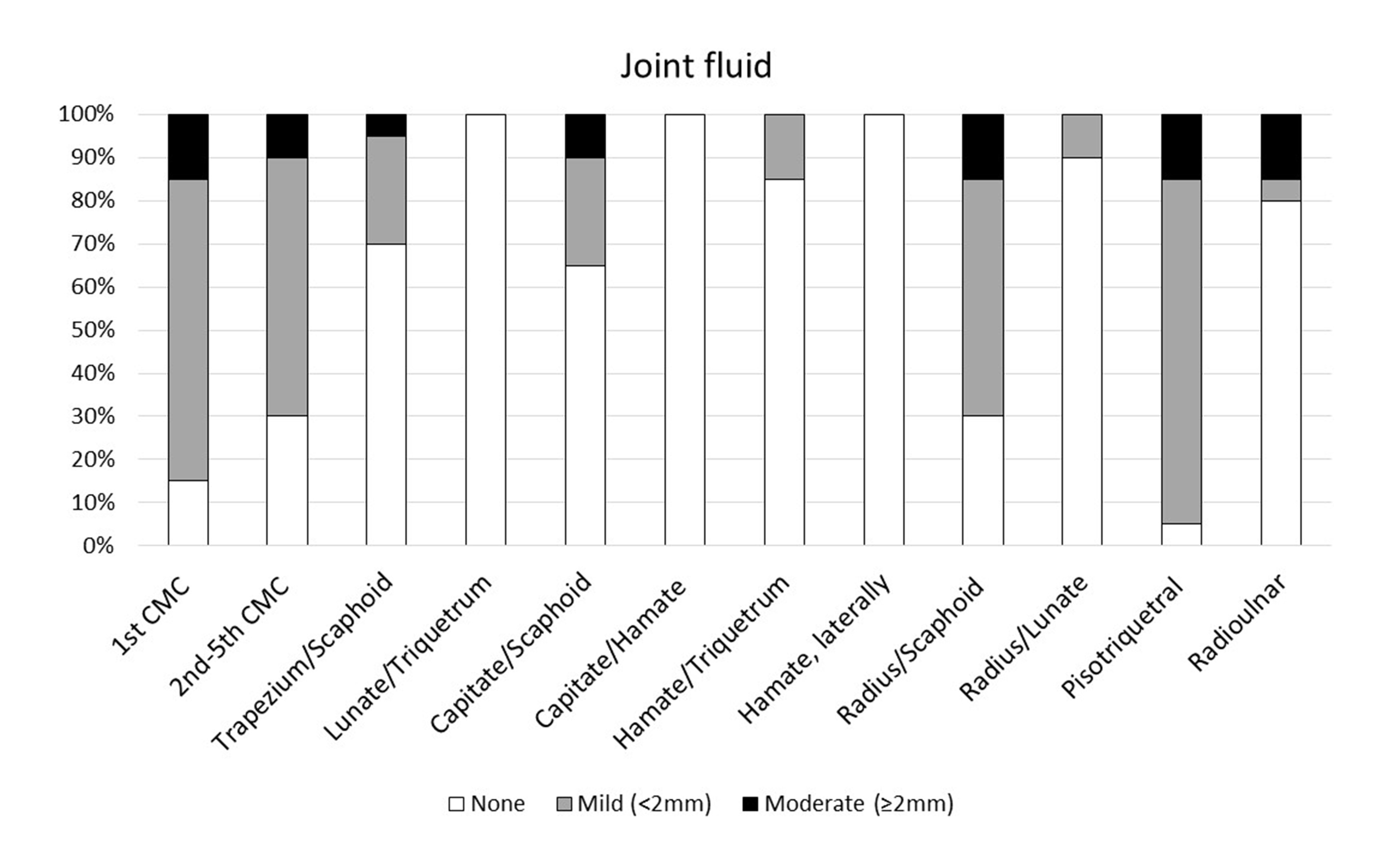

Supplement: Supplementary file 1 — (JPG 965 kb) [file 247_2020_4739_MOESM1_ESM.jpg]

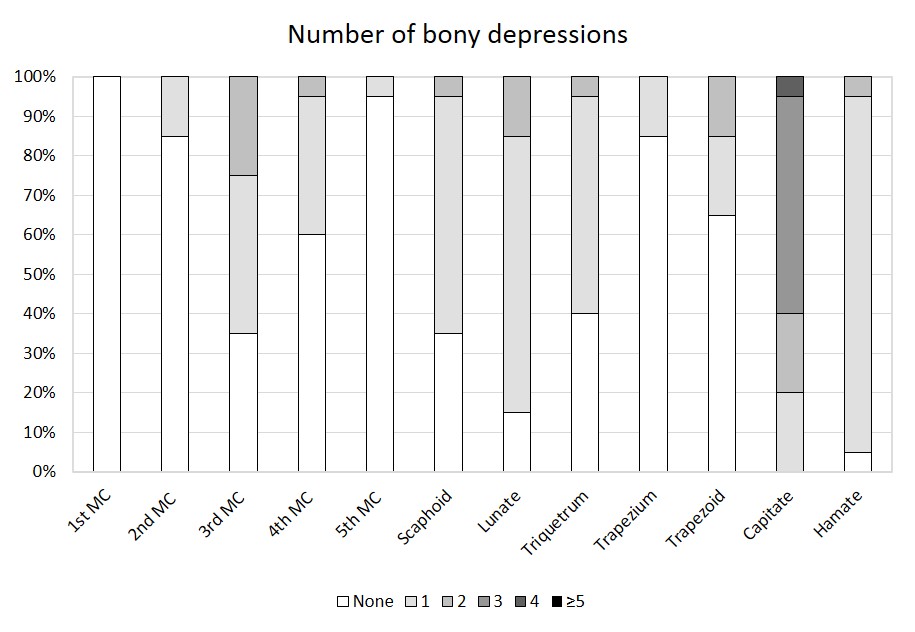

Supplement: Supplementary file 2 — (JPG 74 kb) [file 247_2020_4739_MOESM2_ESM.jpg]

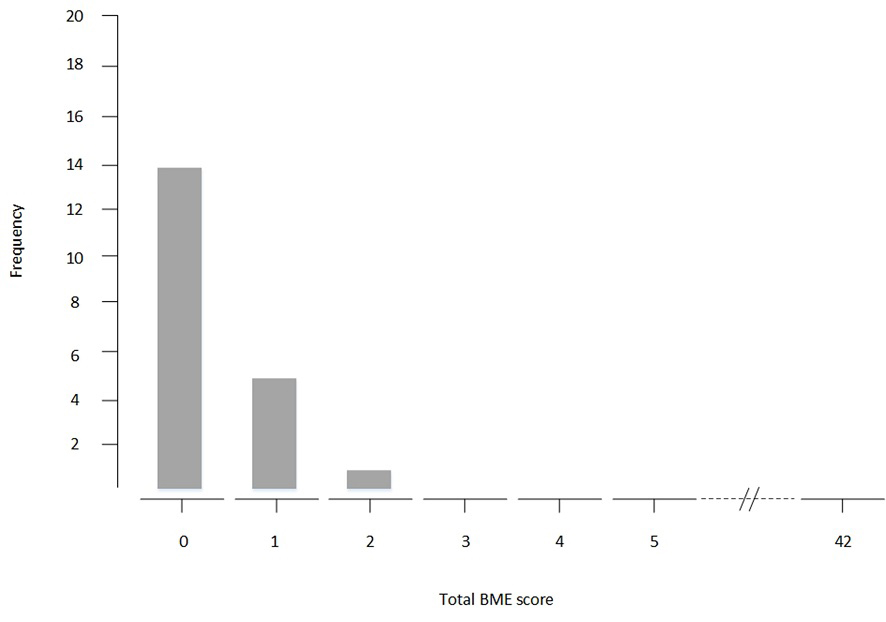

Supplement: Supplementary file 3 — (JPG 79 kb) [file 247_2020_4739_MOESM3_ESM.jpg]

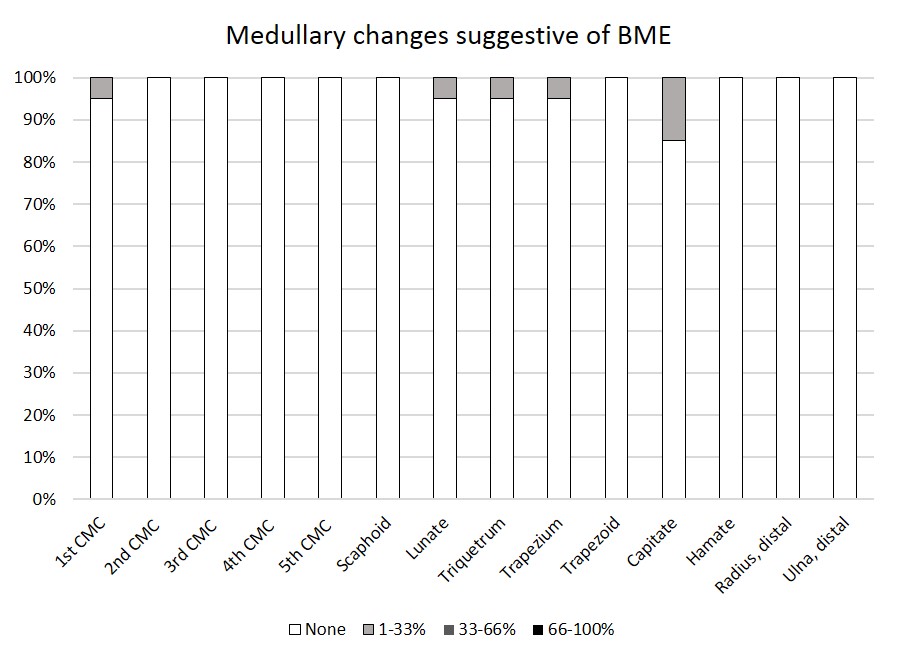

Supplement: Supplementary file 4 — (JPG 85 kb) [file 247_2020_4739_MOESM4_ESM.jpg]
